# ADORA1 is a diagnostic-related biomarker and correlated with immune infiltrates in papillary thyroid carcinoma

**DOI:** 10.7150/jca.50743

**Published:** 2021-05-13

**Authors:** Xu Lin, Zhi-Yong Wang, Gang Xue, Xiao-Jing Qin, Jing-Fang Wu, Geng Zhang

**Affiliations:** 1 Zhangjiakou Key Laboratory of Thyroid Cancer Precision Diagnosis, Hebei North University, Zhangjiakou, 075000, China.; 2 Department of Histology and Embryology, Hebei North University, Zhangjiakou, 075000, China.; 3 Department of Otorhinolaryngology Head and Neck Surgery, Hebei North University, Zhangjiakou, 075000, China.

**Keywords:** papillary thyroid carcinoma, ADORA1, immune infiltrates, biomarker

## Abstract

**Background:** Adenosine A1 Receptor (ADORA1) is an adenosine receptor particularly relevant to the immunomodulatory process of malignant tumors. There are growing evidences that dysregulated overexpression of ADORA1 can promote many types of tumorigenesis. However, the expression and prognostic value and mechanism of ADORA1 in thyroid papillary carcinoma have not been reported.

**Methods:** TCGA, ONCOMINE, UALCAN, cBioPortal, GeneMANIA, LinkedOmics, TIMER, GSCALite, TISIDB and EPIC tools were used in this study.

**Results:** ADORA1 was overexpressed in papillary thyroid carcinoma compared to paracancerous tissue. And ADORA1 was positively correlated with lymph node metastasis as well as pathological stage in PTC. ADORA1 had diagnostic and prognostic value for PTC. The functions of ADORA1 co-expressed genes were mainly enriched in immune response, immune response-regulation signaling pathway, regulation of leukocyte activation and cancer-related pathways. Besides, ADORA1 expression was significantly correlated with tumor-infiltrating cells and immune biomarkers in PTC. Finally, the high expression of ADORA1 was sensitive to JW-55 drug.

**Conclusion:** ADORA1 is a diagnostic and a prognostic biomarker for PTC. The expression of ADORA1 is positively correlated with many immunoregulatory factors in PTC.

## Introduction

Thyroid papillary carcinoma (PTC), originating from thyroid follicular epithelial cells, is a differentiated endocrine malignancy 1. The incidence of PTC has been steadily increasing, and the incidence rate reached 6.20 per 100,000 in females and 1.80 per 100,000 in males on a world-wide scale 2. Patients suffering from PTC with cervical lymph node metastases or distant metastases usually have a poor prognosis, and a small percentage of patients (about 10-15%) may develop into a life-threatening recurrent disease 3-6. Although the evolution of PTC is thought to be influenced by many factors such as genetic factors, hormone levels and ionizing radiation, the specific pathogenesis of PTC is still unclear, especially the underlying mechanism of immune regulation of specific genes 7-9. Vigneri et al. noted that about half of PTC patients were accompanied by immune infiltration of macrophages and T lymphocytes 9. And immunoregulatory targets such as PDL1, PDL2, PD1, LAG-3, TIM-3 were all aberrantly expressed in PTC 9.

Adenosine A1 Receptor (ADORA1) is an adenosine receptor particularly relevant to the immunomodulatory process of malignant tumors, with four family members (ADORA1, ADORA2a, ADORA2b and ADORA3) playing regulatory roles *in vitro* 10-12. Adenosine has been reported to accumulate abnormally in the tumor microenvironment and binds to adenosine receptors to maintain the immune homeostasis in cancers. It has been well documented that abnormally overexpression of ADORA1 can facilitate the malignant progressions of colon cancer 13, kidney cancer 14, breast cancer 15, glioblastoma 16 and leukemia 17. Programmed cell death protein 1 (PD-1) and its ligand (PD-L1) have been previously reported as immunotherapeutic targets for many solid cancers with significant efficacy 18-20. Liu et al demonstrated that silencing ADORA1 expression in human melanoma cell lines significantly increased tumor PD-L1 levels and inhibited T cell-mediated cytotoxicity by conducting T cell-mediated cancer cell-killing assays 11. These evidences all suggest that ADORA1 may mediate the immune microenvironment to influence cancer progression and the regulation of immunity. However, the specific mechanisms by which ADORA1 regulates tumor progression and immune cell infiltration in PTC are unclear.

Herein, we first analyzed the ADORA1 expression in PTC using the TCGA database, Oncomine database, and then tested whether ADORA1 expression in tissue microarrays was consistent with bioinformatics predictions using immunohistochemical staining experiments. ADORA1 gene alterations, functional enrichment analysis, protein-protein interaction networks, single nucleotide variation and ADORA1 associated regulatory factors (kinase, miRNA and transcription factor-target) in PTC were comprehensively evaluated. We analyzed the association between ADORA1 and tumor immune cell infiltration, and the relationship between ADORA1 and immune-related modulators using Tumor Immunoassay Resource (TIMER) and TISIDB tools, respectively. Finally, the relationships between differentially expressed ADORA1 and immune cells in PTC were analyzed using EPIC based on TCGA data.

## Materials and methods

### Data preparation and cBioPortal

Data concerning the ADORA1 mRNA expression profiles across the 512 TC samples and 18 normal tissue were downloaded from The Cancer Genome Atlas (TCGA) database. The OncoPrint of ADORA1and the relationships between the ADORA1 genetic variations and arm-level SCNA cluster assignment, extrathyroidal extension and disease stage were analyzed using the cBioPortal website, according to Papillary Thyroid Carcinoma (TCGA, Cell 2014) data 21. Patients meeting the following criteria were excluded: patients had clinical information but no gene expression data.

### Tissue samples

The PTC tissue microarrays were acquired from Shanghai Outdo Biotech CO., LTD. (NO. HThy-Pap120CS-01, Shanghai, China). Patients meeting the following criteria were excluded: (1) all patient samples in this tissue microarray were pathologically diagnosed as PTC; (2) all patient samples required no additional treatment. And the exclusion criteria were as follows to exclude patients with incomplete information on clinicopathological data.

### UALCAN analysis and Oncomine database analysis

Both the UALCAN 22 and Oncomine 23 databases were online sites where you could acquire the expression, clinical stage, and survival status data of genes. In this study, we first used the UALCAN and Oncomine databases to analyze ADORA1 expression in PTC and paracancerous tissues. The expression of ADORA1 in PTC patients with different stage, gender, age, race and lymph node metastasis were then analyzed. P-value less than 0.05 was considered statistically significant.

### Kaplan Meier plotter analysis and LinkedOmics analysis

The overall survival and relapse free information of ADORA1 were analyzed by Kaplan Meier plotter based on GEO and TCGA data 24. LinkedOmics 25 was a comprehensive web application that could be used to analyze specific genes and their interconnections, functional enrichment analysis and certain regulatory factors in specific cancers. The interconnections, functional enrichment analysis (GO and KEGG analysis) and certain regulatory factors (transcriptional factors, microRNAs and protein kinases) of ADORA1 were analyzed by LinkedOmics.

### Immunohistochemistry

Immunohistochemistry of ADORA1 was performed using a rabbit monoclonal anti-ADORA1 antibody (1:500 dilution, Bioss, bs-6649R).

Concrete procedure was referred to the previous study 26. Briefly, 4 μm PTC tissue microarray sections were dewaxed, hydrated and antigen-repaired before incubation with anti-ADORA1 primary antibody (1:500 dilution) at 4 °C overnight. 120-point PTC tissue chips were then washed with PBS and incubated with broad-spectrum secondary antibodies. Finally, the PTC tissue microarrays were photographed using an Olympus Biomicroscope. And the overall IHC score of ADORA1 in PTC and corresponding paracancerous tissue grading from 1 to 5 was assessed owing to the semi-quantitative immunoreactive score (IRS) scale of Remmele 27. The percentage of tumor cells was scored as follows: 0 (0-5% positive cells); 1 (6-25% positive cells); 2 (26-50% positive cells); 3 (51-75% positive cells); and 4 (76-100% positive cells). Staining intensity was scored as follows: 0 (no staining); 1-2 (weak staining); and >2 (strong staining).

### GeneMANIA analysis and GSCALite analysis

GeneMANIA was an analytical tool to analyze the interconnection of gene clusters 28. Interactions between ADORA1 and its top-four most significantly associated genes were analyzed. The GSCALite website was a multifunctional genomics site that could be used to analyze gene expression, methylation, single nucleotide variation, pathway activity and drug targets in our study with TCGA PTC sample 29. The single nucleotide variation, drug targets and pathway activity of ADORA1, MYBPH, FAM178B, CHI3L1, METTL7B were analyzed using GSCALite tool. p <0.05 was considered statistically significant.

### Immune-related analysis

TIMER, an interactive web server, enabled online analysis of immune cells, immune genes and immunomodulatory factors in a variety of cancers using TCGA database data 30. Analysis of ADORA1 expression in relation to biomarker levels of tumor-infiltrating immune cells using the “correlation” module. TISIDB was a web portal that integrates multiple heterogeneous data types on the interaction of tumor immunomodulatory factors 31. In this experiment, we analyzed the regulatory factors and immune subtype that could be involved in ADORA1 immunoregulation in the PTC. EPIC could complete the estimation of immune cells in cancer samples based on its own algorithm by integrating gene expression in different cancer samples 32. To assess the effect of ADORA1 expression on immune cells, we classified the 512 PTC samples from TCGA into the ADORA1 high expression group (top 256 cases) and the ADORA1 low expression group (low 256 cases). The EPIC application tool and GraphPad Prism 7 were used for immunoassay and results visualization, respectively. p <0.05 was considered statistically significant.

## Results

### Expression and prognostic value of ADORA1 in PTC

**Figure 1:**
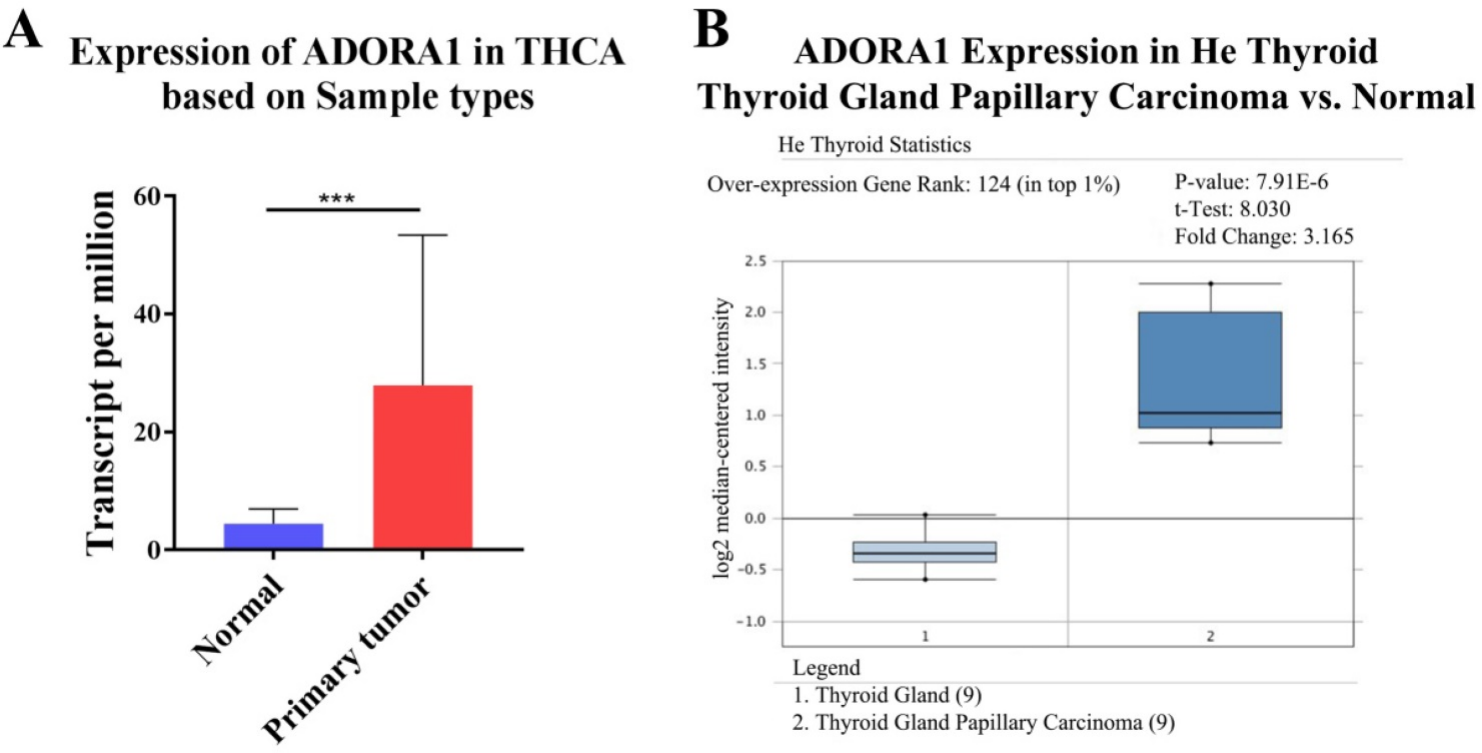
The transcription levels of ADORA1 in thyroid cancer. Compared with normal samples, ADORA1 mRNA was overexpressed in PTC (A) UALCAN database and (B) ONCOMINE database. *** p<0.001.

**Figure 2:**
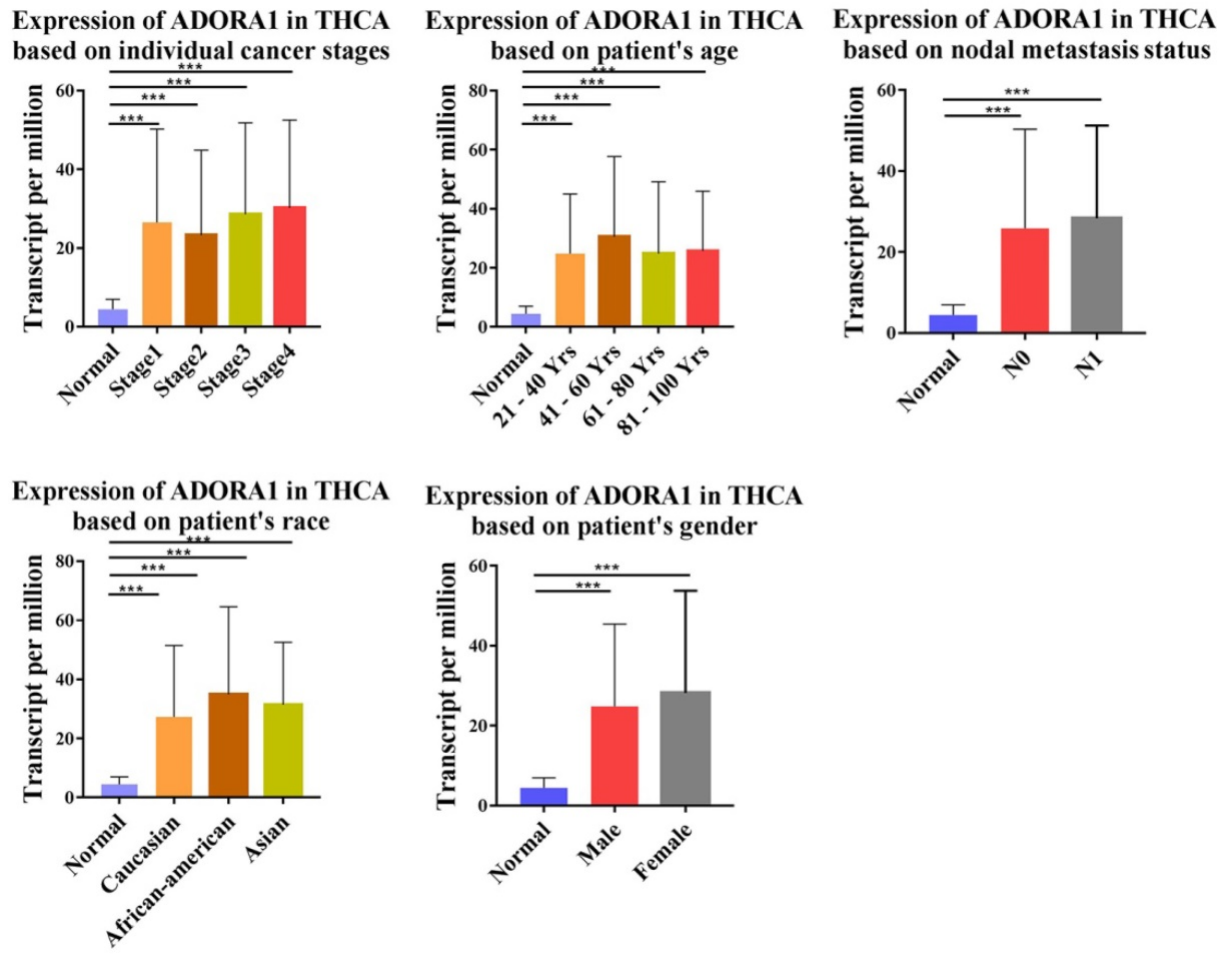
Correlation between ADORA1 expression and tumor stage in TC patients (UALCAN). Compared with normal tissues, ADORA1 expression was positively correlated with the nodal metasis, clinical stage. *p<0.05, **p<0.01, ***p<0.001.

**Figure 3:**
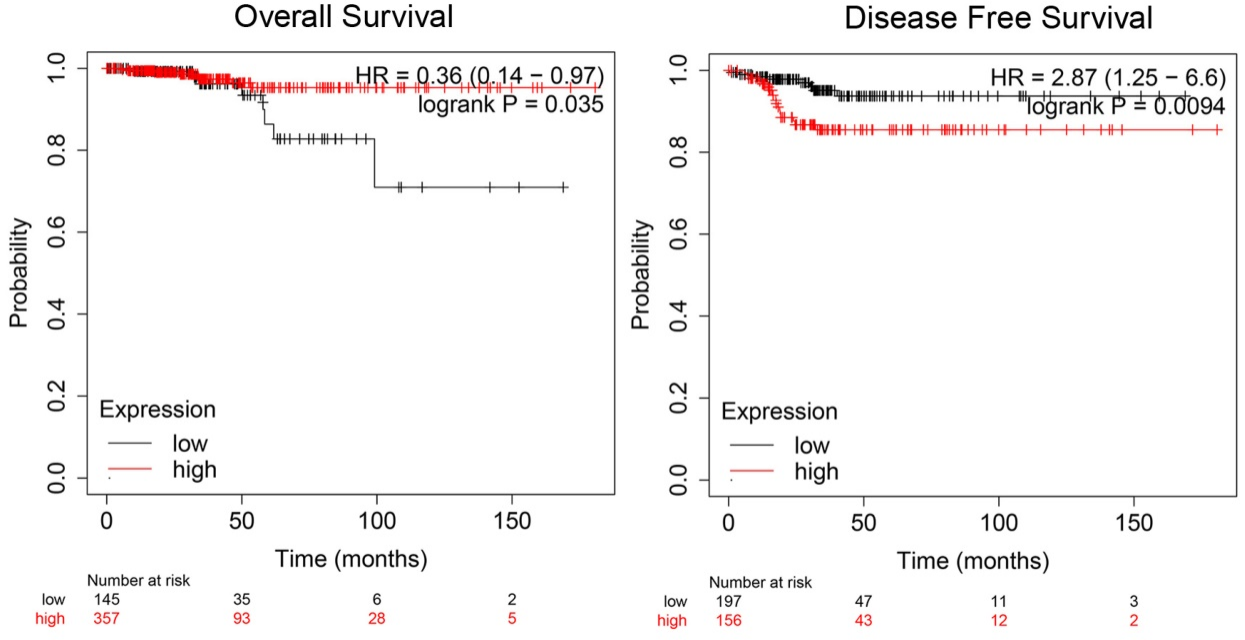
Prognostic value of mRNA expression of ADORA1 in thyroid cancer patients (Kaplan-Meier Plotter).

**Figure 4:**
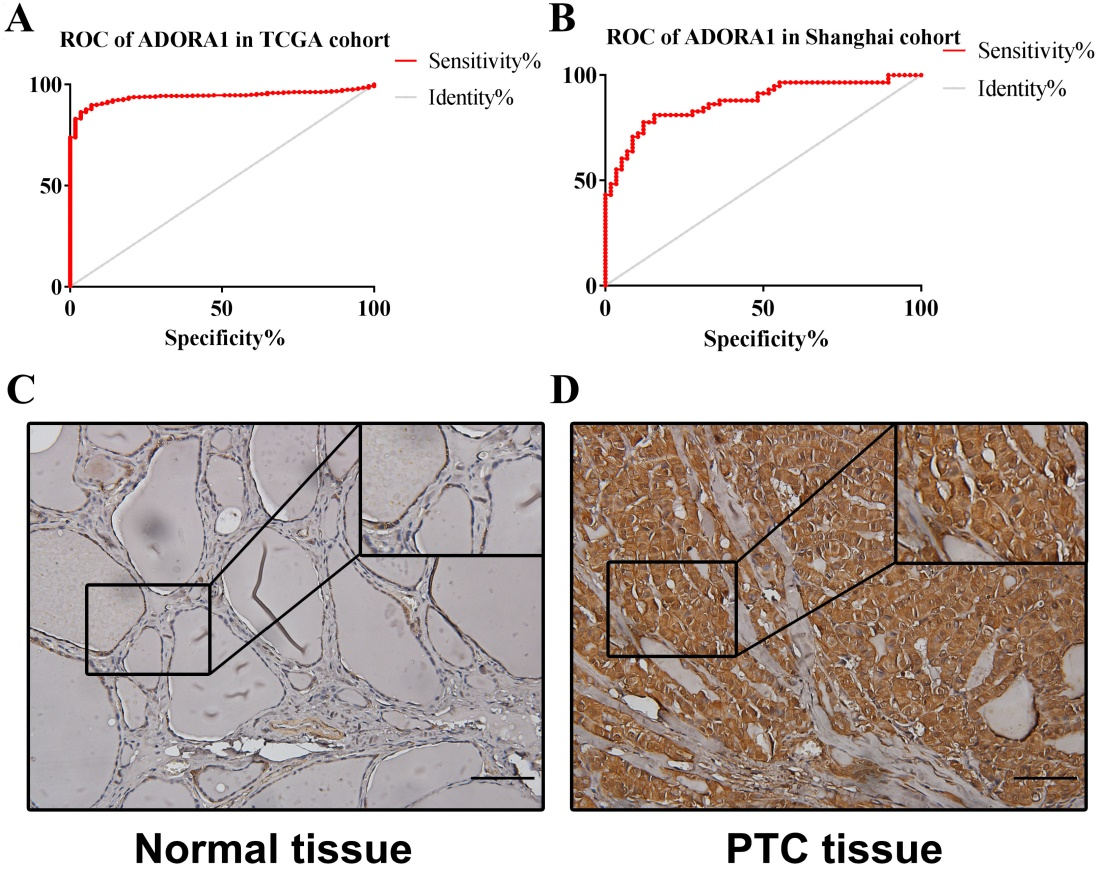
ROC curves and immunohistochemistry results of ADORA1. (A) AUC of ADORA1 in TCGA cohort was 0.9408 (95% CI: 0.9209-0.9606), p <0.0001. (B) AUC of ADORA1 in Shanghai cohort was 0.9064 (95% CI: 0.8546-0.9581), p <0.0001. (C-D) ADORA1 proteins were higher in PTC tissues, compared to tumor-adjacent tissues, Scale bar: 20 µm.

To illuminate the role of ADORA1 in papillary thyroid cancer, we first evaluated its expression, diagnostic value and prognostic value in patients with PTC. Data in the TCGA database and the Oncomine database revealed that mRNA expression of ADORA1 were significantly higher in papillary thyroid cancer tissues than that in normal tissues (Figure 1). Then, we analyzed the expression of ADORA1 with the tumor stage, patients' age, patients' race, patients' gender and nodal metastasis status for thyroid carcinoma. Regardless of tumor stage, race, gender, age and nodal metastasis status, ADORA1mRNA levels in PTC tissues were significantly higher than in normal thyroid tissues (Figure 2). The prognostic value of ADORA1 mRNA expression levels in thyroid cancer was conducted utilising Kaplan-Meier Plotter. The results revealed that high expression of ADORA1 (HR=2.87, 95% CI: 1.25-6.6, and p=0.0094) was associated with a worse disease free survival in the thyroid carcinoma patients while high expression of ADORA1 (HR=0.36, 95% CI: 0.14-0.97, and p=0.035) was associated with a longer overall survival (Figure 3). Besides, immunochemical staining experiment of PTC tissue microarrays (Shanghai cohort) revealed that ADORA1 was overexpressed in PTC tissues compared to paracancerous tissues and ROC curves were performed to assess the diagnostic role of ADORA1 in PTC (Figure 4). And ADORA1 expression was positively correlated with pathological stage (p = 0.00001, r = 0.632) and lymph node metastasis (p = 0.01, r = 0.64) in Shanghai cohort (Table 1). As shown in Figure 4, the AUC of ADORA1 in TCGA cohort was 0.9408 (95% CI: 0.9209- 0.9606, p <0.0001) and the AUC of ADORA1 in Shanghai cohort was 0.9064 (95% CI: 0.8546-0.9581, p <0.0001). Thus, ADORA1 can be considered as a potential diagnostic indicator of PTC.

### Genomic alterations and enrichment analysis of ADORA1 correlated genes in PTC

**Figure 5:**
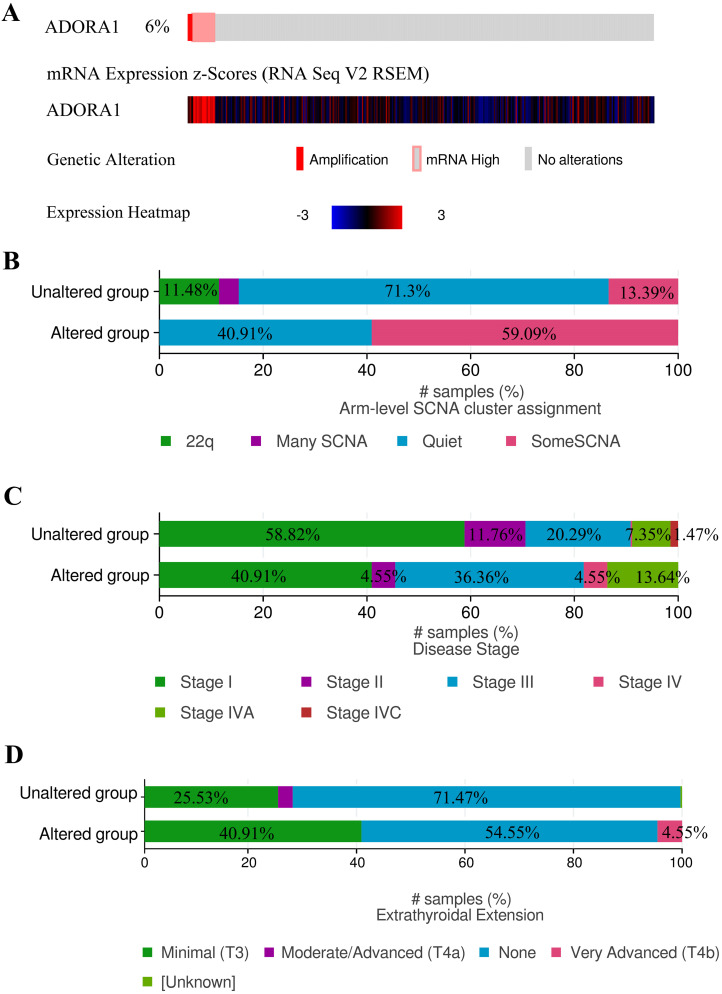
ADORA1 genomic alterations in PTC. (A) OncoPrint of ADORA1 alterations in PTC cohort. (B) Distribution of ADORA1 CNV frequency in arm-level SCNA cluster assignment. (C) Distribution of ADORA1 CNV frequency in different stages. (D) Distribution of ADORA1 CNV frequency in different extrathyroidal extension.

Due to the significance of ADORA1 in PTC, the types and frequency of ADORA1alterations in PTC were analyzed by cBioPortal tool according to DNA sequencing data from PTC patients. ADORA1 was altered in 22 of 338 (6%) PTC patients. These alterations involved mRNA up-regulation in 18 cases (4.6%) and amplification in 4 cases (1.4%) (TCGA, Cell 2014) (Figure 5A). Besides, the frequency distribution of ADORA1 CNV patients in arm-level SCNA cluster assignment, different stage and extrathyroidal extension were presented in Figure 5B-5D, suggesting the early-event and disease progression of ADORA1 CNV alteration in PTC.

**Figure 6:**
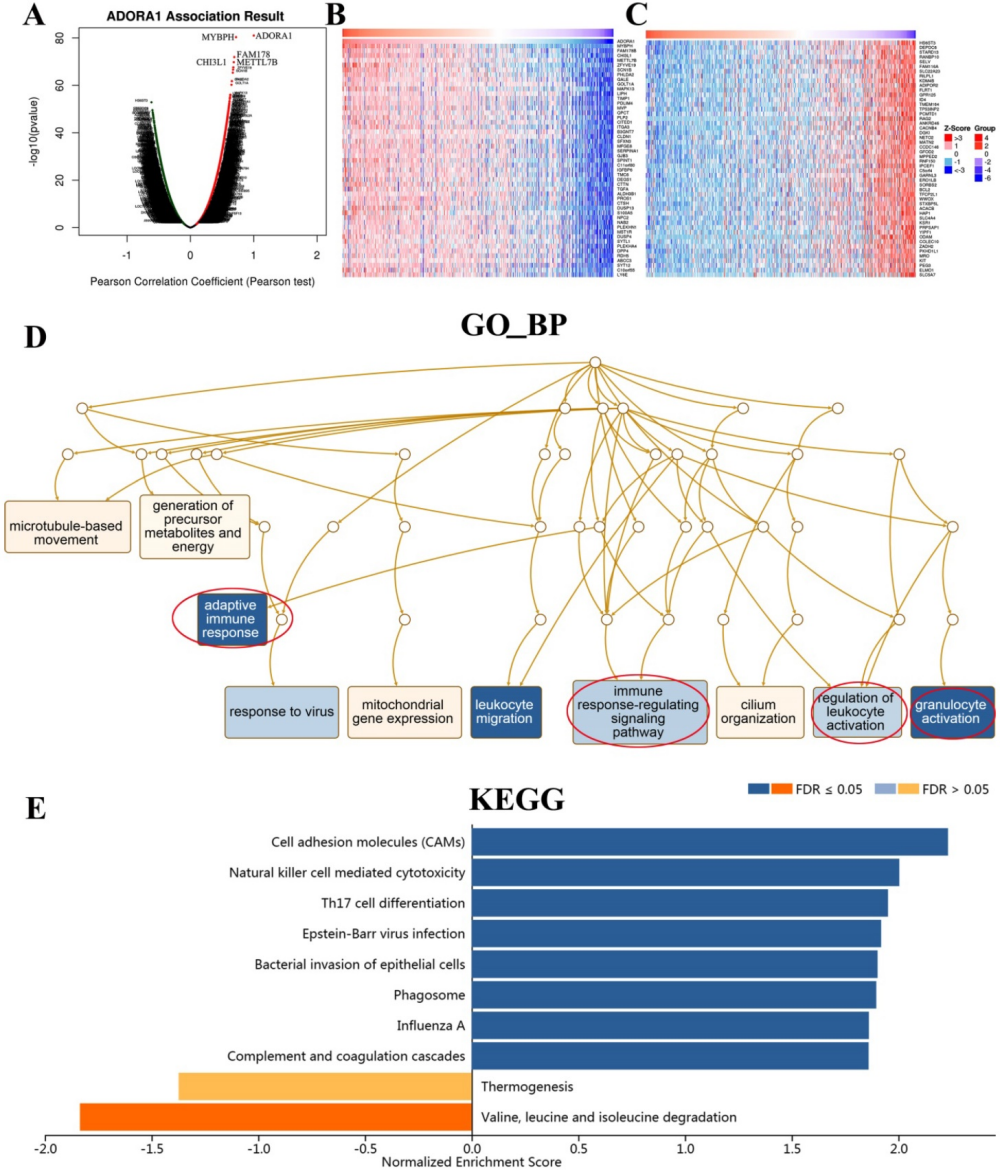
ADORA1 co-expression genes in PTC (LinkedOmics). (A) The genes positively and negatively correlated with ADORA1 in PTC. (B) The top-50 gene positively correlated with ADORA1 in PTC in heat maps. (C) The top-50 gene negatively correlated with ADORA1 in PTC in heat maps. (D) Biological process analyse and KEGG pathways of ADORA1 in TCGA cohort (E).

To gain insight into the biological significance and function of ADORA1 in PTC, multiple modules of LinkedOmics were used to examine ADORA1 co-expression patterns, functional enrichment analysis and regulators in the PTC cohort. As shown in Figure 6A, 5713 genes (dark red dots) were shown positive correlations with ADORA1, whereas 7050 genes (dark green dots) were shown negative correlations with ADORA1 (false discovery rate, FDR < 0.05). In addition, the top 50 important genes positively and negatively associated with ADORA1 in the PTC were presented in Figure 6B and 6C, respectively. Moreover, ADORA1 and the top 4 significant genes, including MYBPH (cor=0.0.71, p=4.86e-81), FAM178B (cor=0.69, p = 1.22e-72), CHI3L1 (cor = 0.68, p = 1.74e-70) and METTL7B (cor = 0.67, p = 2.68e-680), positively correlated with ADORA1 in PTC, were considered as the hub genes (Supplementary Figure 1).

Significant Gene Ontology (GO) term annotation showed that ADORA1 correlated genes mainly participated in adaptive immune response, immune response-regulation signaling pathway, regulation of leukocyte activation, granulocyte activation, cilium organization, mitochondrial gene expression, response to virus, microtubule-based movement and generation of precursor metabolites and energy (Figure 6D). The KEGG pathway indicated that ADORA1 and its related genes were primarily associated with cell adhesion molecules (CAMs), natural killer cell-mediated cytotoxicity, Th17 cell differentiation, epstein-barr virus infection, bacterial invasion of epithelial cells, phagosome, influenza A, complement and coagulation cascades and valine, leucine and isoleucine degradation (Figure 6E).

### Kinase, miRNA or transcription factor targets of ADORA1 in PTC

To further explore the regulators of ADORA1 in PTC, we performed an analysis of kinases, miRNAs and transcription factors of ADORA1 gene (Table 2). For kinase networks of ADORA1, only one kinase target of ADORA1 was identified (Kinase_STK11) according to the LinkedOmics database. To probe the function of kinase STK11 in more details, we next constructed PPI networks of STK11. And the results showed that these genes were primarily involved in the regulation of insulin receptor signaling pathway, cell cycle arrest, negative regulation of cell cycle, cellular response to peptide hormone stimulus, protein serine/threonine kinase activity, protein kinase activator activity (Supplementary Figure 2). For miRNAs, MIR-221 and MIR-222 were enriched by GSEA of ADORA1 co-expressed genes. Interestingly, the functions of the genes of MIR-222 network were mainly involved in protein insertion into mitochondrial membrane involved in apoptotic signaling pathway, positive regulation of protein insertion into mitochondrial membrane involved in apoptotic signaling pathway, regulation of chromatin silencing, regulation of chromosome organization, negative regulation of gene expression, epigenetics, protein insertion into mitochondrial membrane, regulation of protein insertion into mitochondrial membrane involved in apoptotic signaling pathway (Supplementary Figure 3). Moreover, the transcripttion factors (GGGNNTTTCC_V$NFKB_Q6_01, V$AP1_Q4_01, V$AP1_C, V$AP1_Q4, V$PEA3_Q6, V$NFKB_Q6, V$NFKAPPAB_01, TGASTMAGC_V$NFE2_01, TGANTCA_V$AP1_C, V$STAT_01, V$AP1_Q6, V$TEF1_Q6, V$BACH1_01, V$PAX5_02, V$AP1FJ_Q2, V$AP1_Q2_01, V$AP1_Q2, TTCYNRGAA_V$STAT5B_01, V$BACH2_01, V$NFKB_Q6_01, RYTTCCTG_V$ETS2_B, V$SREBP1_Q6 and V$ELF1_Q6) of ADORA1 were shown in Table 2. And the functions of these transcription factors were primarily enriched in plasma lipoprotein particle assembly, protein-lipid complex assembly, macrophage-derived foam cell differentiation, foam cell differentiation, protein-lipid complex subunit organization, sequence-specific cDNA binding, regulation of plasma lipoprotein particle levels (Supplementary Figure 4).

### The correlation between ADORA1 expression and immune biomarkers of ADORA1 in PTC

**Figure 7:**
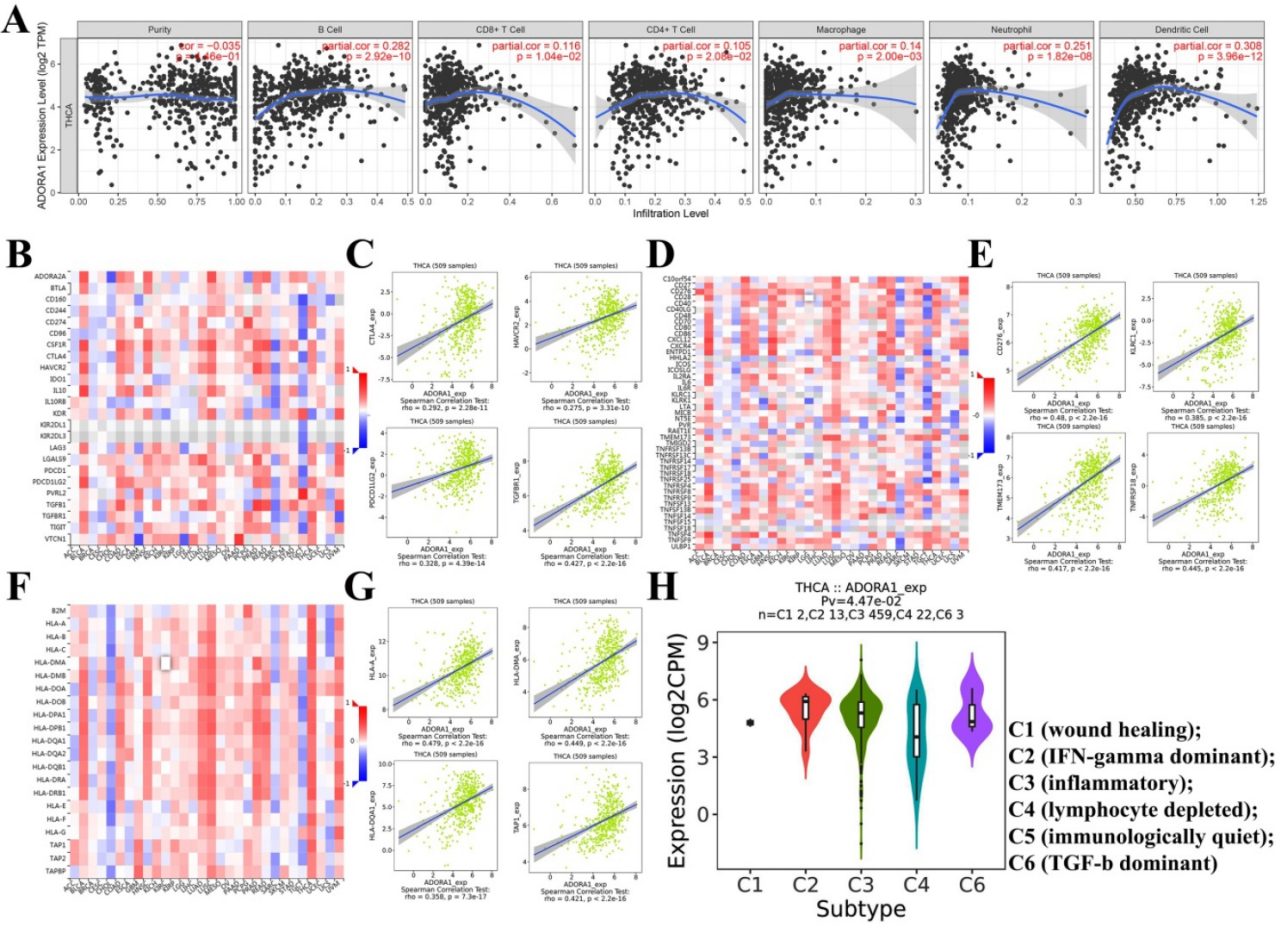
Correlation of ADORA1 expression with immune cell and immunomodulators in thyroid cancer. (A) ADORA1 expression in the PTC were positively associated with B cell (Cor=0.282, P=2.92e-10), CD8+ T cells (Cor=0.116, P=1.04e-02), CD4+ T cells (Cor = 0.105, P = 2.08e-02), Macrophage (Cor = 0.14, P = 2.00e-03), Neutrphils (Cor = 0.251, P = 1.82e-08) and Dendritic cells(Cor = 0.308, P = 3.96e-12). (B) Relations between the immunoinhibitors and ADORA1 expression. (C) 4 immunoinhibitors were correlated with ADORA1 expression. (D) Relations between the immunostimulators and ADORA1 expression. (E) 4 immunostimulators were correlated with ADORA1 expression. (F) Relations between the MHC molecules and ADORA1 expression. (G) 4 MHC molecules were correlated with ADORA1 expression. (H) ADORA1 expression was related to immune subtypes in PTC including wound healing, IFN-gamma dominant, inflammatory, lymphocyte depleted, and TGF-b dominant.

**Figure 8:**
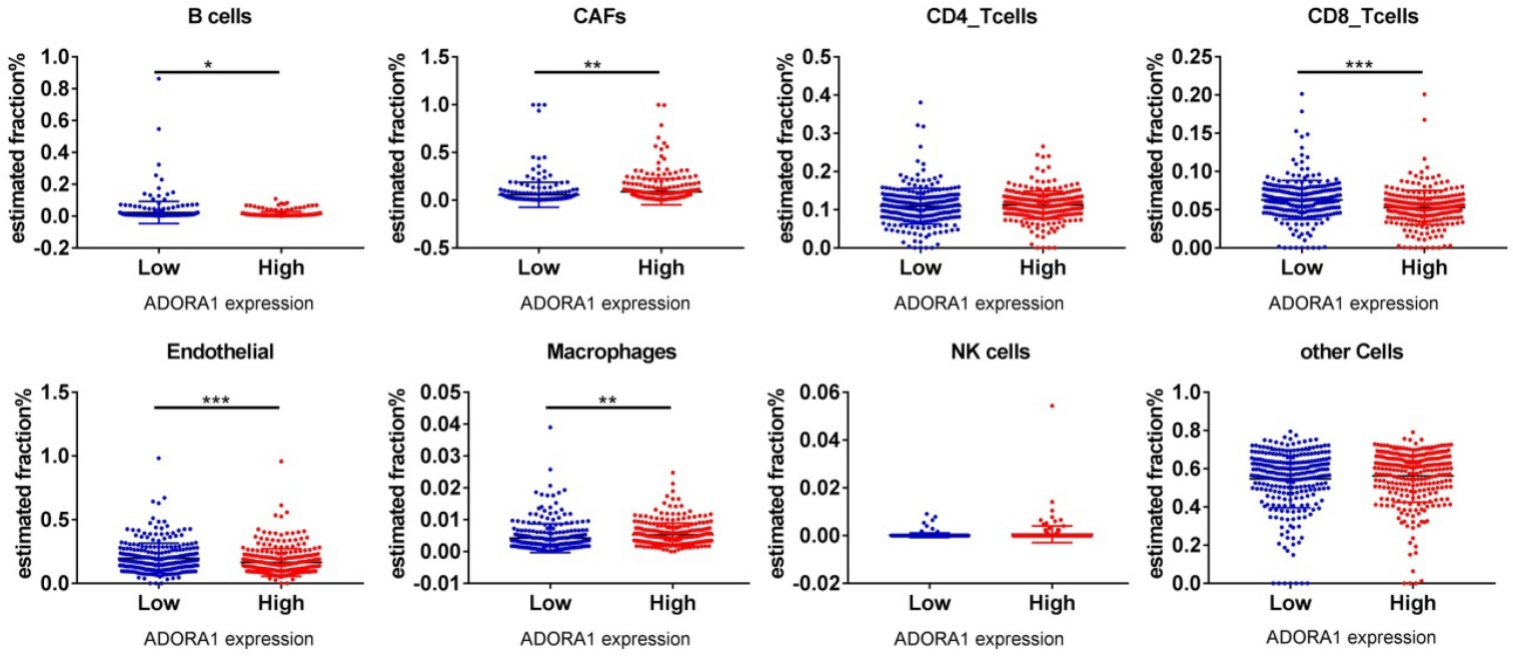
Different immune cells expression in ADORA1 low group and ADORA1 high group analyzing by online tools EPIC. *P < 0.05, **P < 0.01, ***P < 0.001.

It has been previously documented that ADORA1 may mediate the immune microenvironment to influence cancer progression and immune regulation 18-20. Thus, the correlations of ADORA1 expression in PTC with immune cells and biomarkers were investigated using the TIMER and TISIDB tools. As shown in Figure 7A, ADORA1 expression in the PTC was positively associated with B cell (Cor=0.282, P=2.92e-10), CD8+ T cells (Cor=0.116, P=1.04e-02), CD4+ T cells (Cor = 0.105, P = 2.08e-02), Macrophage (Cor = 0.14, P = 2.00e-03), Neutrphils (Cor = 0.251, P = 1.82e-08) and Dendritic cells(Cor = 0.308, P = 3.96e-12). Besides, we performed an analysis of the association between ADORA1 expression and immunomodulators using the TISIDB database. Figure 7B demonstrated correlations between ADORA1 expression and immune inhibitors. The immune inhibitors showed strong correlations with ADORA1 expression including CTLA4 (Spearman: ρ = 0.292, P = 2.28e-11), TGFBR1 (Spearman: ρ = 0.427, P < 2.2e-16), HAVCR2 (Spearman: ρ = 0.275, P = 3.31e-10) and PDCD1LG2 (Spearman: ρ = 0.328, P = 4.39e-14) in PTC (Figure 7B-7C). For immunostimulators, ADORA1 expression was positively correlated with CD276 (Spearman: ρ = 0.48, P < 2.2e-16), KLRC1 (Spearman: ρ = 0.385, P < 2.2e-16), TMEM173 (Spearman: ρ = 0.417, P < 2.2e-16) and TNFRSF18 (Spearman: ρ = 0.445, P < 2.2e-16) in PTC (Figure 7D-7E). Figure 7F-7G revealed correlations between ADORA1 expression and MHC molecules. There were positive correlations between ADORA1 expression and HLA-A (Spearman: ρ = 0.479, P < 2.2e-16), HLA-DMA (Spearman: ρ = 0.449, P < 2.2e-16), HLA-DQA1 (Spearman: ρ = 0.358, P = 7.3e-17) and TAP1 (Spearman: ρ = 0.421, P < 2.2e-16) in PTC. In addition, ADORA1 expression was related to immune subtypes in PTC including wound healing, IFN-gamma dominant, inflammatory, lymphocyte depleted, and TGF-b dominant (Figure 7H). To understand the relationships between ADORA1 expression and immune cell markers in more detail, the relationship between ADORA1 expression and many of these immune markers were analyzed. As shown in Table 3, remarkable correlations were found between ADORA1 expression and the expression of CD2, CD115, IRF5, CD11b, HLA-DPB1, HLA-DQB1, HLA-DRA, HLA-DPA1, BDCA-1, STAT4, STAT6, STAT5A and FOXP3. Finally, the relationships between different ADORA1 expression and immune cells in PTC patients were analyzed using the EPIC application. The results showed that B cells (P = 0.018), CAFs (P=0.0039), CD8 T cells (P <0.0001), Endothelial cells (P=0.0006), Macrophage cells (P =0.0018) were main immune cells affected by different ADORA1 expression (Figure 8).

### The genetic variation, interrelationship, cancer pathway and drug susceptibility analysis of 5 hub genes

**Figure 9:**
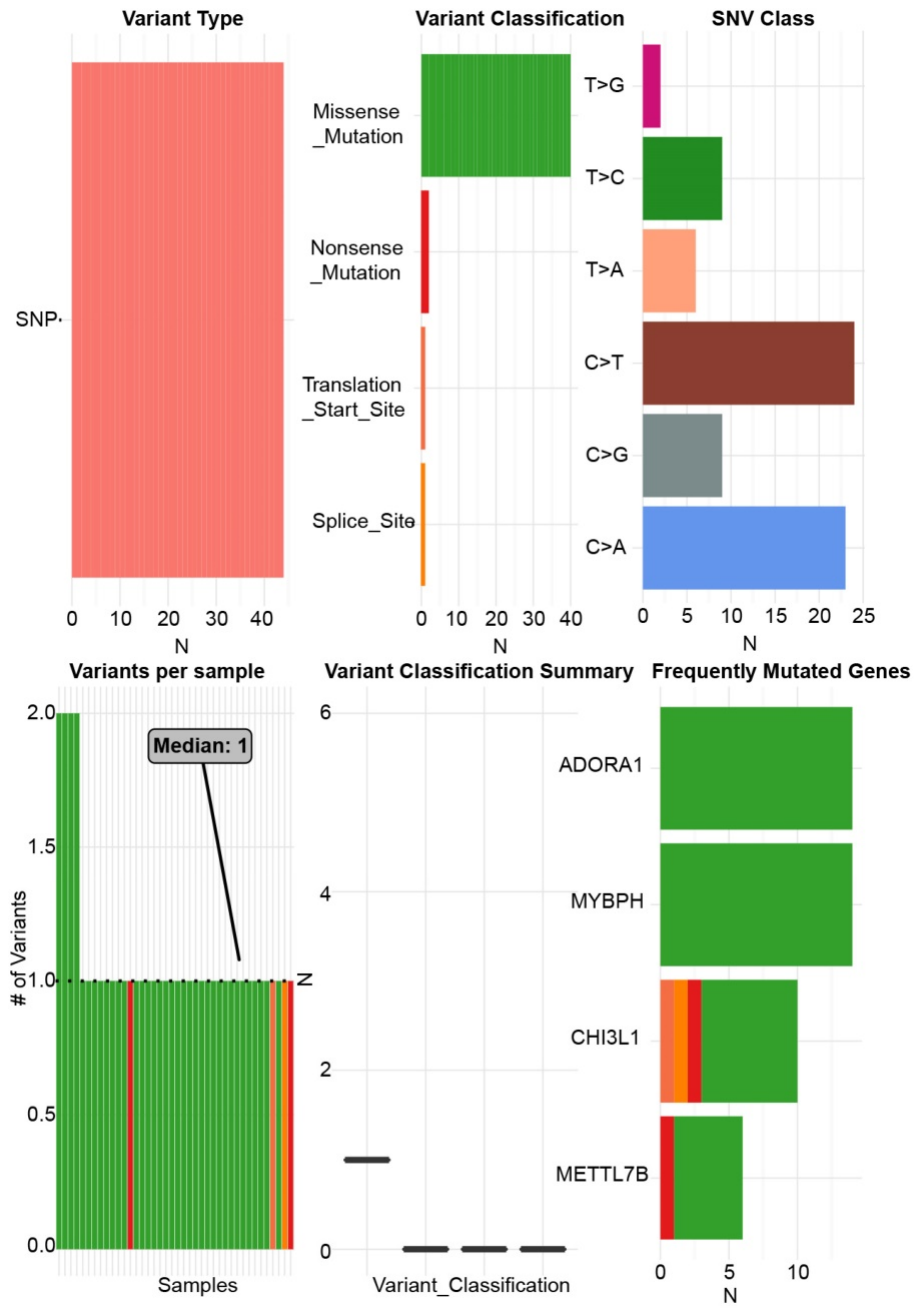
The single nucleotide variation analysis of ADORA1, MYBPH, FAM178B, CHI3L1 and METTL7B.

**Figure 10:**
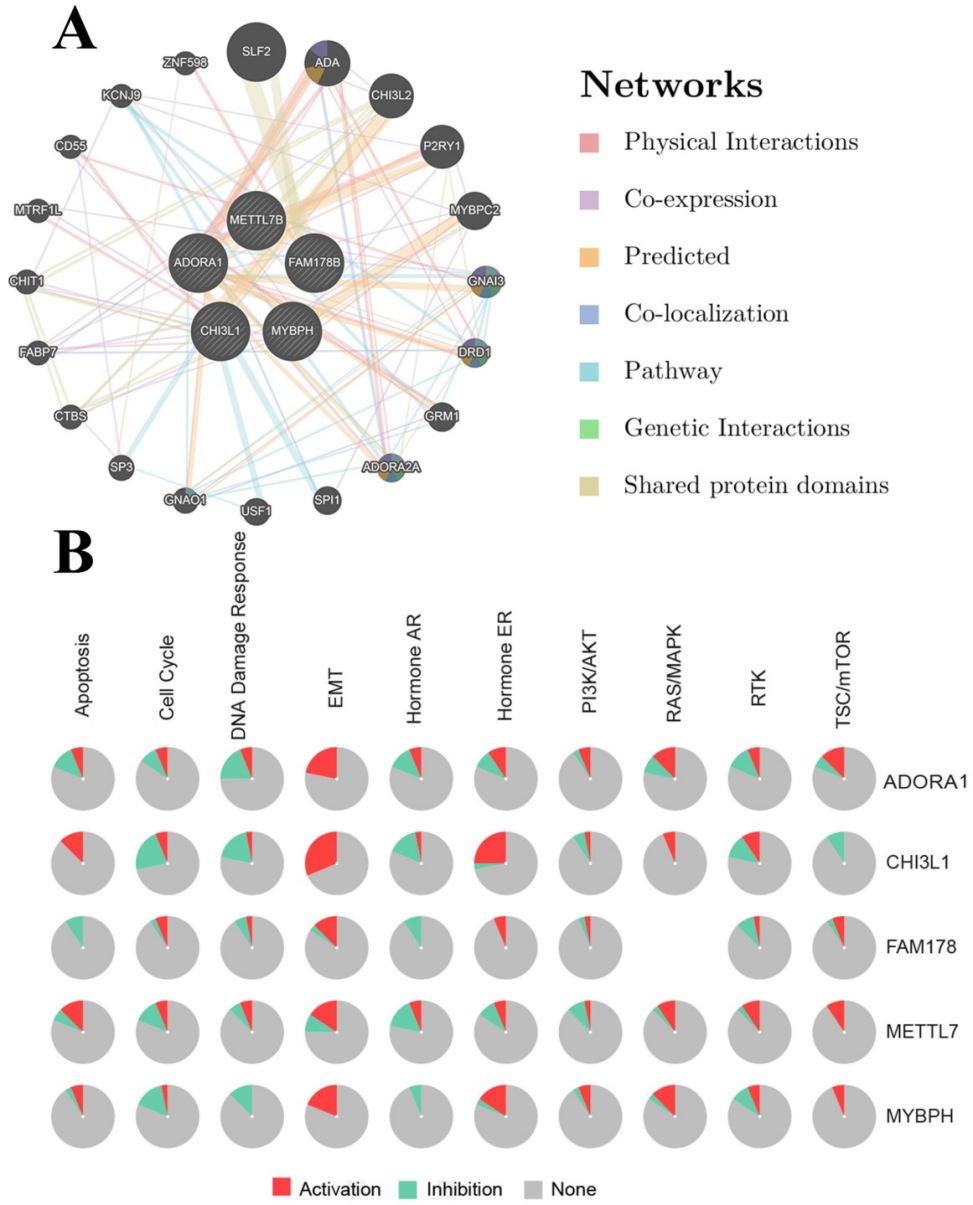
PPI network of ADORA1 (A) and the role of ADORA1 in the cancer related pathways (B).

**Figure 11:**
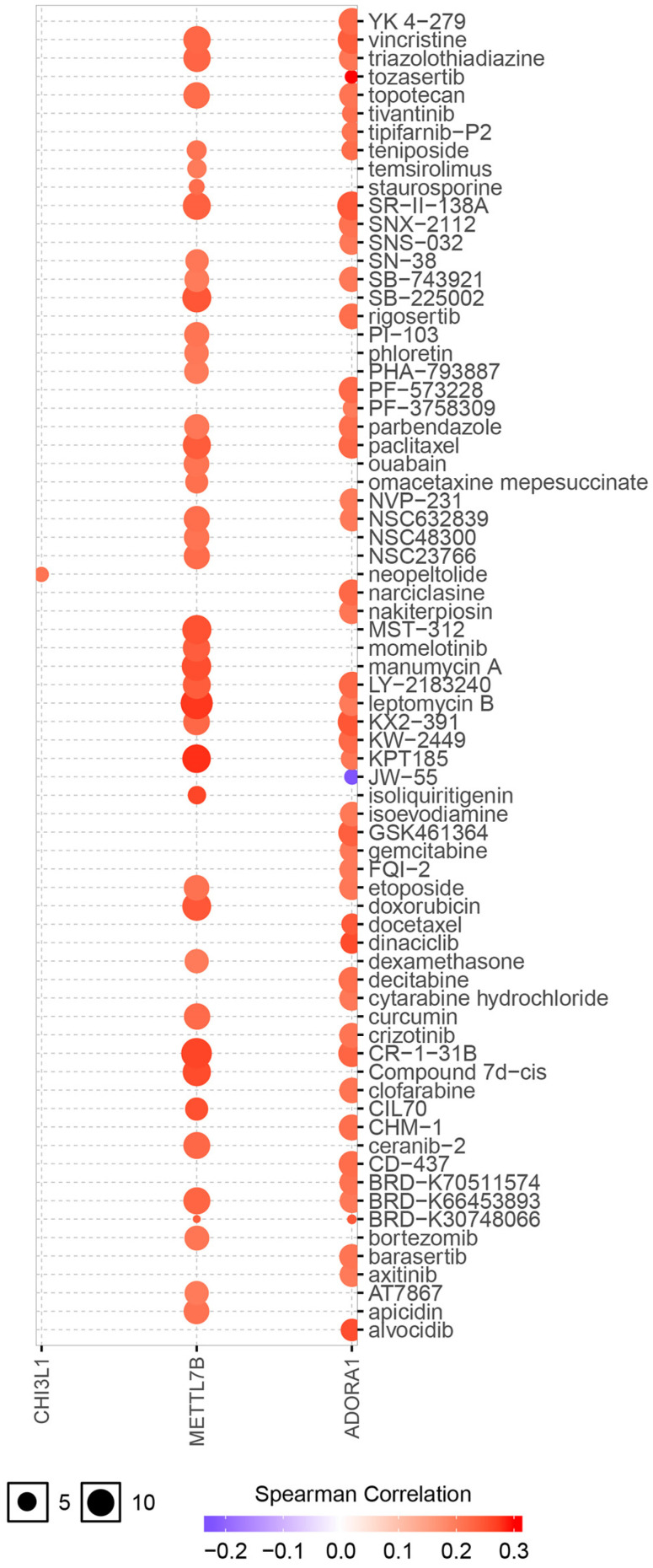
Drug susceptibility analysis of hub genes according to Therapeutics Response Portal (CTRP) data. A positive correlation between gene expression and drug indicates that the gene has an antagonistic effect on the drug.

ADORA1 and the top 4 significant genes, including MYBPH, FAM178B, CHI3L1 and METTL7B were selected as the hub genes for genetic variation, interrelationship, cancer pathway and drug susceptibility analysis. For genetic variation analysis, waterfall plot showed the mutation distribution and SNV classification types of 5 hub genes (Figure 9). And potential interactions between these 5 genes were displayed using GeneMANIA analysis (Figure 10A). Then, we used the GSCALite tool to analyze the potential roles of these 5 genes in the classical cancer pathways. The results showed that these five genes, especially ADORA1, could activate EMT, Hormone ER, PI3K/AKT, RAS/MAPK, TSC/mTOR pathways, and inhibit RTK, Hormone AR, DNA Damage Response, Cell Cycle, Apoptosis pathways to exert regulatory effects in cancer process (Figure 10B). For drug susceptibility analysis, through the GSCALite website we analyzed the relationship between ADORA1 expression and multiple drug sensitivities according to Therapeutics Response Portal (CTRP). The results showed that high expression of METTL78B was resistant to 41 drugs or small molecules. And the high expression of ADORA1 was also resistant to 46 drugs or small molecules. Besides, we found that high expression of ADORA1 was sensitive to JW-55 drugs (Figure 11). Therefore, this provided additional targets for PTC immunotherapy.

## Discussion

Adenosine was well recognized as a potent modulator of both innate and acquired immunity 33. To date, there were a total of four types of adenosine G-protein-coupled receptors, which were ADORA1, ADORA2a, ADORA2b and ADORA3 34. Hasko et al. noted that ADORA1 not only had the highest affinity for adenosine, but was also abundantly expressed in immune cells 35. Moreover, a growing number of reports indicated that ADORA1 could influence the development, progression and metastasis of various kinds of cancers by mediating immune processes. However, the function of ADORA1 in PTC remains unclear. In other words, our study was the first one to investigate the roles of ADORA1 in PTC.

We first examined the expression of ADORA1 in PTC according to the data from TCGA database and the Oncomine database. The results showed that ADORA1 mRNA levels were up-regulated in PTC compared to paracancerous tissues. And there were significant correlations between ADORA1 expression and the clinical stage, metastasis and survival analyses of PTC patients. To verify the bioinformatics predictions regarding the ADORA1 gene, we performed immunohistochemistry experiments on PTC tissue microarrays containing 58 pairs of tissues. As shown by the immunohistochemistry results, ADORA1 was overexpressed in PTC and had positive correlations with pathological stage and lymph node metastasis in Shanghai cohort (Table 1). Dysregulation of ADORA1 overexpression can promote the development of multiple solid tumors. Previous reports pointed out that ADORA1 antagonists could effectively reduce breast cancer cell line Mcf-7 cell proliferation 15. And ADORA1 could regulate the apoptotic effect of breast cancer cells by regulating p53 expression 36. In our study, the AUC of ADORA1 in TCGA cohort was 0.9408 and the AUC of ADORA1 in Shanghai cohort was 0.9064. Thus, ADORA1 can be considered as a potential diagnostic indicator of PTC and may play a crucial role in PTC.

Next, we queried the genes significantly correlated with ADORA1 and their functions in the PTC. GO term annotations showed that the fuctions of ADORA1 correlated genes mainly were involved in adaptive immune response, immune response-regulation signaling pathway, regulation of leukocyte activation, granulocyte activation, cilium organization, mitochondrial gene expression, response to virus, microtubule-based movement and generation of precursor metabolites and energy. And the KEGG pathway data indicated that ADORA1 and its related genes were primarily associated with cell adhesion molecules(CAMs), natural killer cell-mediated cytotoxicity, Th17 cell differentiation, epstein-barr virus infection, bacterial invasion of epithelial cells, phagosome, influenza A, complement and coagulation cascades and valine, leucine and isoleucine degradation. These results all suggested that ADORA1 might have a modulatory effect on cancer progression, especially immunomodulation. Besides, our data showed that ADORA1 could activate EMT, Hormone ER, PI3K/AKT, RAS/MAPK, TSC/mTOR pathways and inhibit RTK, Hormone AR, DNA Damage Response, Cell Cycle, Apoptosis pathways to exert regulatory effects on the cancer process. Sitkovsky et al pointed out that adenosine receptors on the surface of immune cells, especially T lymphocytes, could be activated rapidly and abundantly to regulate immune function when under oxygen-deficient environment 37. Moreover, ADORA1 exerted a modulatory effect on cancer cell proliferation and differentiation by manipulating the MAPK signaling pathway 38. Consequently, the functions of ADORA1 and related genes were primarily involved in tumor immunomodulation and cancer-related pathways, suggesting that ADORA1 may mediate the tumorigenesis and progression of PTC.

Genomic instability and mutations greatly increase the chances of tumorigenesis, while kinases and their associated regulators help stabilize and repair genomic DNA that has been mutated 39. We found significant ADORA1 gene CNV alterations in PTC patients with clinical stage III-IV and extrathyroidal extension. This suggested that ADORA1 gene CNV changes can contribute to the generation of PTC. To further explore the possible mechanism in PTC, we analyzed the kinase, miRNA or transcription factor of ADORA1 using the LinkedOmics database. STK11, a kinase involved in the regulation of ADORA1, was aberrantly expressed in various tumors and played a role in promoting cancer cell adhesion and angiogenesis 40. Besides, LKB1 has been used for immunotherapy 41. In this study, regulators of ADORA1 networks were mainly riched in adaptive immune response, immune response-regulation signaling pathway, regulation of leukocyte activation, granulocyte activation, microtubule-based movement, cell adhesion molecules (CAMs), natural killer cell-mediated cytotoxicity, Th17 cell differentiation and several cancer-related pathways. These suggested that ADORA1 can exert a pro-tumorigenic effect on PTC by modulating these regulators.

Previous study proved that silencing ADORA1 expression significantly increased tumor PD-L1 levels and inhibited T-cell-mediated cytotoxicity in human melanoma cell lines 11. And ADORA1 exerted immunoprotective effects on the organism by activating the immune response. Sitkovsky indicated that ADORA1 on endothelial cells and neutrophils could be activated by adenosine to enhance the ability of cell chemotaxis and release reactive oxygen species to participate in the immune response 37. And there was growing evidence that ADORA1 was strongly associated with immunity. Here, the relationships between ADORA1 expression levels and immune cells and immune markers were analyzed comprehensively. And remarkable correlations were found between ADORA1 expression and the expression of CD2, CD115, IRF5, CD11b, HLA-DPB1, HLA-DQB1, HLA-DRA, HLA-DPA1, BDCA-1, STAT4, STAT6, STAT5A and FOXP3. In addition, the relationships between different ADORA1 expression and immune cells in PTC patients were analyzed using the EPIC application, and we found that different levels of ADORA1 expression was associated with B cells, CAFs, CD8 T cells, endothelial cells and macrophages. Finally, we found that high expression of ADORA1 was sensitive to JW-55 drug. Thus, ADORA1 may play an essential role in the immune microenvironment of PTC, and ADORA1 may be an important immune checkpoint in PTC.

To summarize, ADORA1 was overexpressed in PTC and can be a diagnostic and a prognostic factor. The functions of ADORA1 and associated genes were mainly involved in adaptive immune response, immune response-regulation signaling pathway, regulation of leukocyte activation, cell adhesion molecules (CAMs), natural killer cell-mediated cytotoxicity, Th17 cell differentiation and cancer-related pathways. ADORA1 expression was significantly correlated with immune cells and immune markers. These results all suggest that ADORA1 may play an oncogenic role in PTC.

## Supplementary Material

Supplementary figures.

## Figures and Tables

**Table 1:** ADORA1 expression and clinicopathological characteristics of 58 specimens in Shanghai corhort

Characteristics	Total	ADORA1 expression		*P* value	χ^2^ value	Correlation
		Low	High			r value
**Age (years)**				0.074	3.194	
<45	24	9	15			
≥45	34	22	12			
Gender				0. 5	3.587	
Female	13	6	7			
Male	45	25	20			
Pathological stage				0.00001	23.161	0.632
Low (I + II)	37	28	9			
High (III +IV)	21	3	18			
Lymph node metastasis				0.01	9.757	0.640
Negative	32	22	10			
Positive	26	9	17			

Statistical analyses were performed by the Pearson χ^2^ test.

**Table 2:** The Kinase, miRNA and transcription factor-target networks of ADORA1 in Thyroid carcinoma (LinkedOmics)

Enriched Category	Geneset	LeadingEdgeNum	FDR
Kinase Target	Kinase_STK11	13	0.02804
miRNA Target	ATGTAGC,MIR-221,MIR-222	45	0.045
Transcription Factor Target	GGGNNTTTCC_V$NFKB_Q6_01	50	0
	V$AP1_Q4_01	71	0.0045693
	V$AP1_C	68	0.0049847
	V$AP1_Q4	66	0.0054001
	V$PEA3_Q6	91	0.0055385
	V$NFKB_Q6	89	0.0059816
	V$NFKAPPAB_01	70	0.0061715
	TGASTMAGC_V$NFE2_01	55	0.0074015
	TGANTCA_V$AP1_C	243	0.0076155
	V$STAT_01	55	0.0078093
	V$AP1_Q6	63	0.0081232
	V$TEF1_Q6	51	0.00818
	V$BACH1_01	64	0.0083078
	V$PAX5_02	5	0.0087825
	V$AP1FJ_Q2	61	0.0089724
	V$AP1_Q2_01	67	0.010263
	V$AP1_Q2	58	0.010904
	TTCYNRGAA_V$STAT5B_01	98	0.013817
	V$BACH2_01	66	0.014308
	V$NFKB_Q6_01	64	0.014539
	RYTTCCTG_V$ETS2_B	295	0.018277
	V$SREBP1_Q6	61	0.022198
	V$ELF1_Q6	83	0.022431

**Table 3:** Correlation analysis between ADORA1 and gene biomarkers of immune cells in THCA (TIMER)

Immune cells	Biomarkers	None		Purity	
		Cor	P-value	Cor	P-value
CD8+ T cell	CD8A	0.134	2.5e-03	0.134	3.11e-03
	CD8B	0.136	2.07e-03	0.136	2.66e-03
T cell (general)	CD3D	0.283	9.45e-11	0.283	1.83e-10
	CD3E	0.278	2.19e-10	0.275	6.27e-10
	CD2	0.31	8.97e-13	0.309	3.19e-12
B cell	CD19	0.107	1.61e-02	0.101	2.63e-02
	CD79A	0.153	5.54e-04	0.145	1.34e-03
Monocyte	CD86	0.281	1.35e-10	0.278	4.20e-10
	CD115 (CSF1R)	0.316	3.93e-13	0.316	8.16e-13
TAM	CCL2	0.231	1.54e-07	0.235	1.52e-07
	CD68	0.278	1.67e-10	0.276	5.20e-10
	IL10	0.106	1.63e-02	0.106	1.88e-02
M1 Macrophage	INOS (NOS2)	-0.073	9.82e-02	-0.068	1.33e-01
	IRF5	0.363	2.61e-17	0.366	6.94e-17
	COX2(PTGS2)	0.284	7.81e-11	0.278	4.18e-10
M2 Macrophage	CD163	0.162	2.49e-04	0.157	5.21e-04
	VSIG4	0.239	5e-08	0.241	6.93e-08
	MS4A4A	0.185	2.76e-05	0.179	7.11e-05
Neutrophils	CD66b (CEACAM8)	0.186	2.32e-05	0.195	1.42e-05
	CD11b (ITGAM)	0.374	2.26e-18	0.376	7.41e-18
	CCR7	0.189	1.89e-05	0.187	3.15e-05
Natural killer cell	KIR2DL1	-0.074	9.49e-02	-0.065	1.49e-01
	KIR2DL3	0.11	1.27e-02	0.114	1.14e-02
	KIR2DL4	-0.006	8.88e-01	-0.011	8.08e-01
	KIR3DL1	0.011	8e-01	0.003	9.48e-01
	KIR3DL2	0.055	2.16e-01	0.055	2.26e-01
	KIR3DL3	0.002	9.67e-01	0.003	9.44e-01
	KIR2DS4	0.007	8.75e-01	0.007	8.75e-01
Dendritic cell	HLA-DPB1	0.397	0e+00	0.399	4.01e-20
	HLA-DQB1	0.38	0e+00	0.38	3.53e-18
	HLA-DRA	0.404	0e+00	0.403	1.57e-20
	HLA-DPA1	0.399	0e+00	0.399	4.49e-20
	BDCA-1 (CD1C)	0.362	3.29e-17	0.37	2.94e17
	BDCA-4 (NRP1)	-0.021	6.41e-01	-0.029	5.28e-01
	CD11c (ITGAX)	0.268	9.11e-10	0.263	3.51e-09
Th1	T-bet (TBX21)	0.119	7.18e-03	0.116	1.03-02
	STAT4	0.373	0e+00	0.367	5.35e-17
	STAT1	0.379	8.53e-19	0.383	1.56e-18
	IFN-g (IFNG)	0.211	1.48e-06	0.209	3.08e-06
	TNF-a (TNF)	0.243	2.9e-08	0.241	7.47e-08
Th2	GATA3	0.09	4.24e-02	0.09	3.39e-03
	STAT6	0.352	2.94e-16	0.347	3.09e-15
	STAT5A	0.333	1.25e-14	0.329	8.92e-14
	IL13	0.092	3.84e-02	0.085	6.07e-02
Tfh	BCL6	-0.016	7.2e-01	-0.032	4.76-e-01
Th17	STAT3	0.254	6.78e-09	0.249	2.58e-08
	IL17A	0.09	4.33e-02	0.098	2.96e-02
Treg	FOXP3	0.328	4.11e-14	0.339	1.46e-14
	CCR8	0.26	2.64e-09	0.268	1.80e-09
	STAT5B	0.074	9.65e-02	0.067	1.40e-01
	TGFb (TGFB1)	0.226	2.54e-07	0.219	1.01e-06
T cell exhaustion	PD-1 (PDCD1)	0.058	1.89e-01	0.064	1.60e-01
	CTLA4	0.275	2.76e-10	0.276	5.31e-10
	LAG3	0.211	1.59e-06	0.209	3.24e-06
	TIM-3 (HAVCR2)	0.272	5.12e-10	0.269	1.46e-09
	GZMB	0.143	1.23e-03	0.14	1.91e-03
