# Supplementary material for: ADORA1 is a diagnostic-related biomarker and correlated with immune infiltrates in papillary thyroid carcinoma
Source: J Cancer. 2021 May 13;12(13):3997–4010. doi: 10.7150/jca.50743 (PMC8176250; doi:10.7150/jca.50743)
Supplement: Supplementary file 1 — Supplementary figures. [file jcav12p3997s1.pdf]

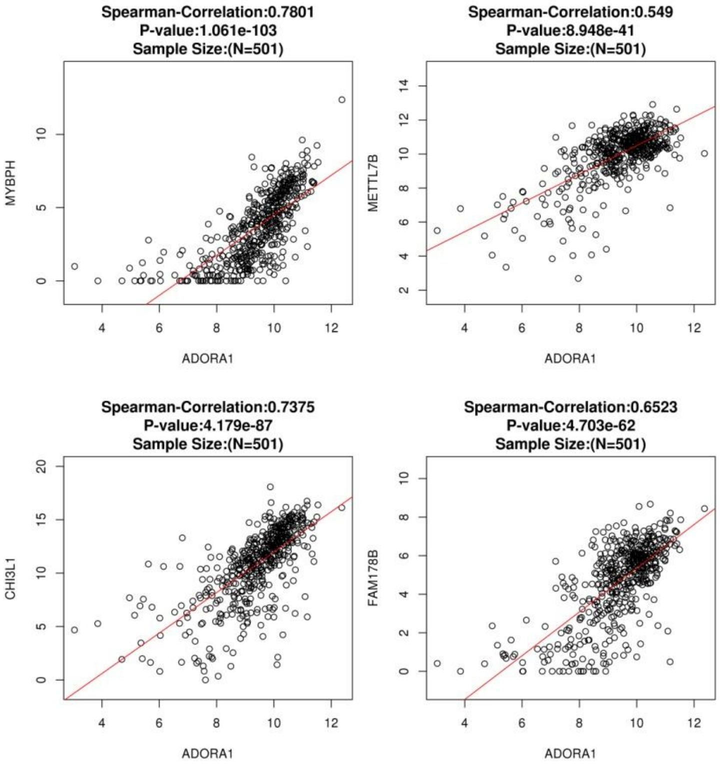

Figure S1. The correlation between the hub genes and ADORA1 in PTC (LinkedOmics).

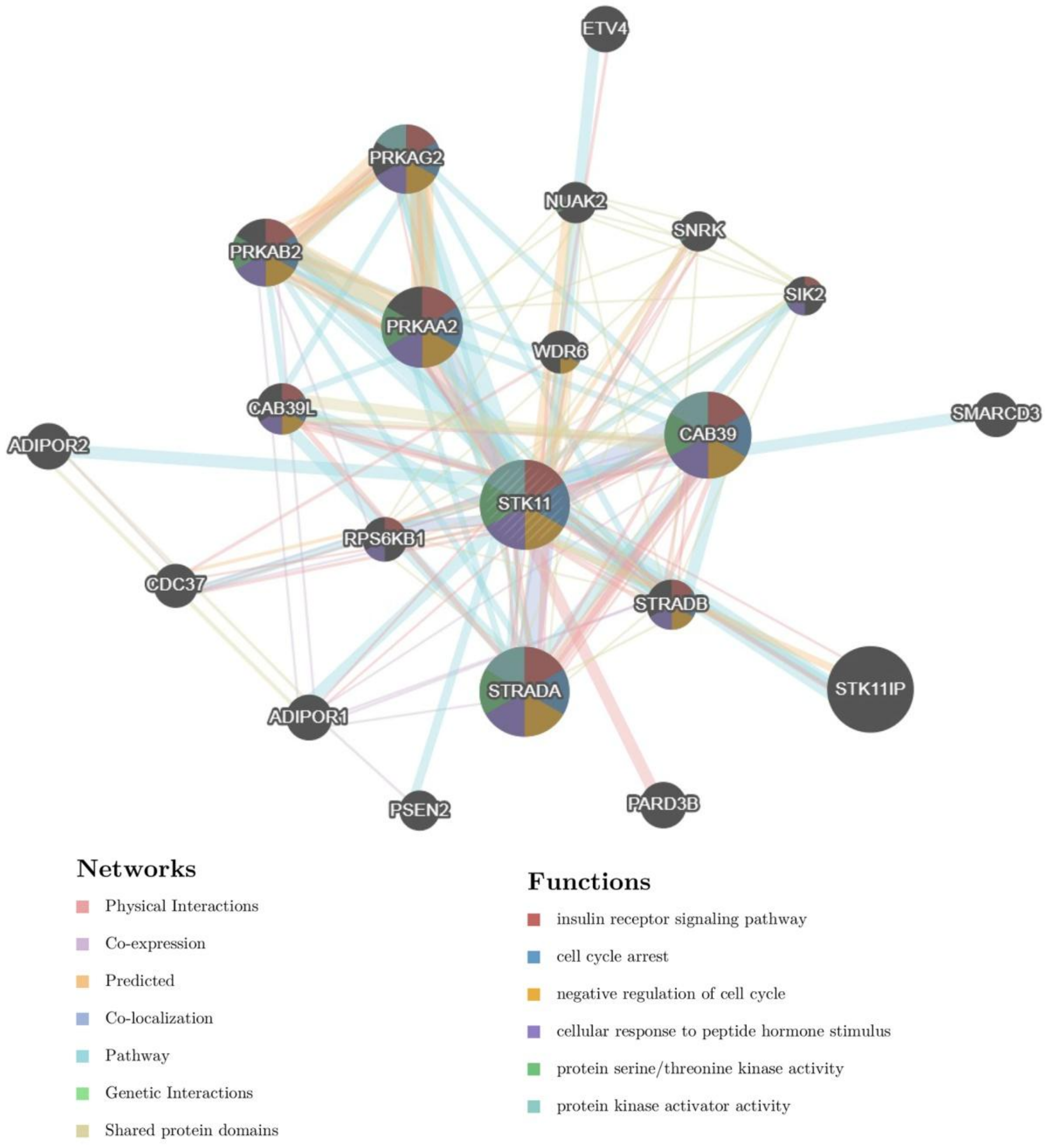

Figure S2. PPI network of STK11 kinase-target networks (GeneMANIA).

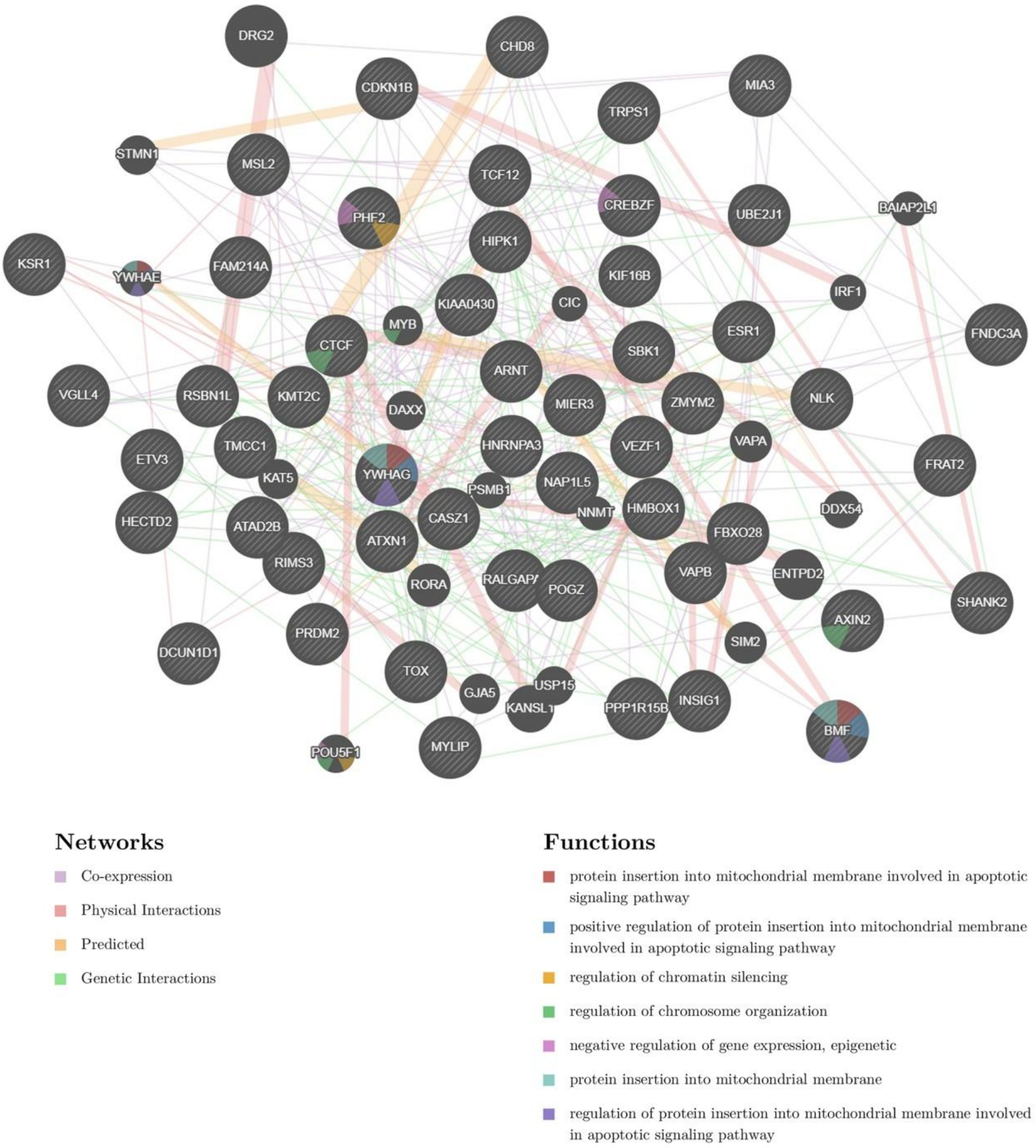

Figure S3. PPI network of MIR-222-target networks (GeneMANIA)

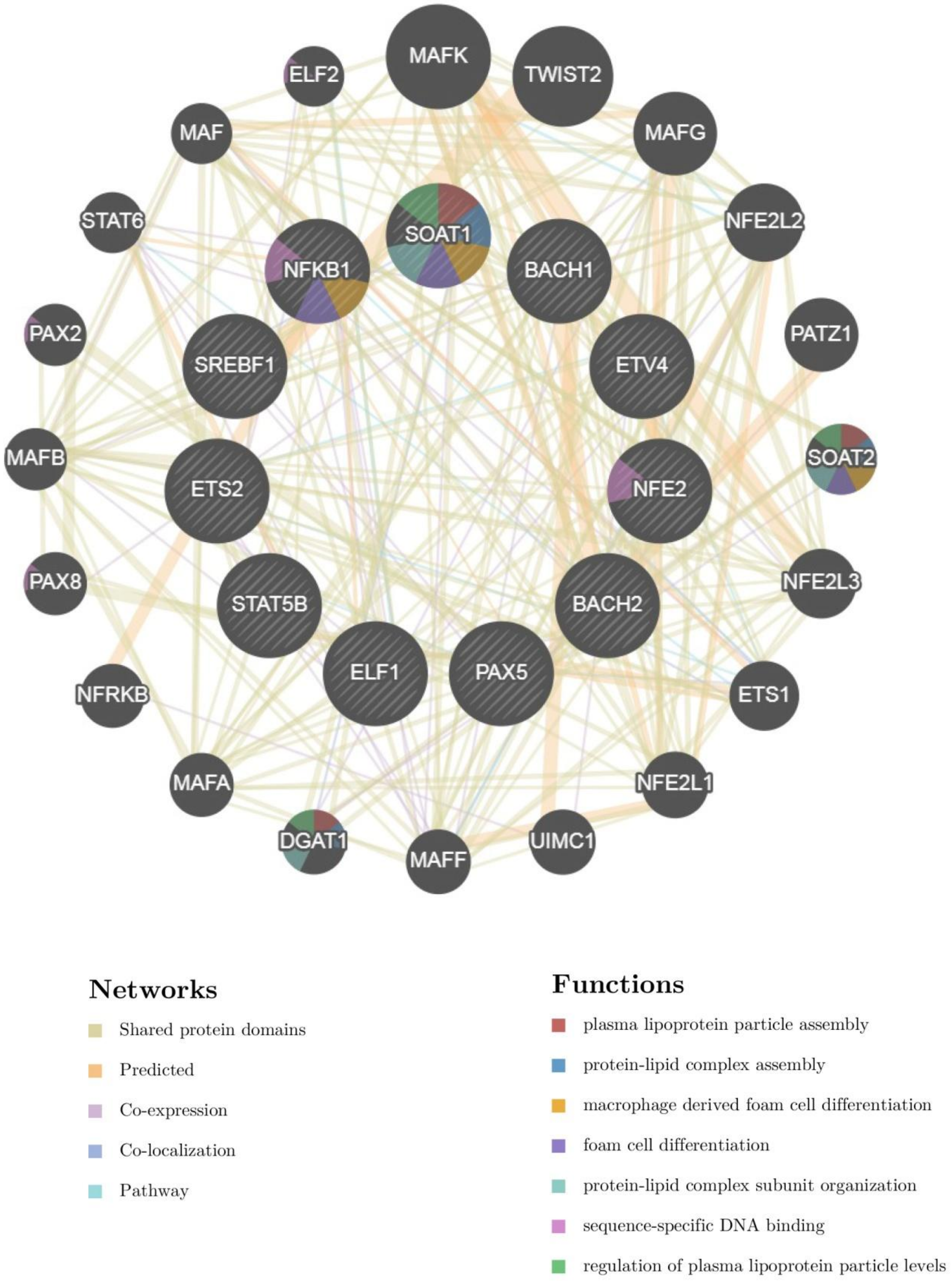

Figure S4. PPI network of transcription factors networks of ADORA1 in PTC (GeneMANIA).
